# Viruses in sanctuary chimpanzees across Africa

**DOI:** 10.1002/ajp.23452

**Published:** 2022-11-03

**Authors:** Emily Dunay, Leah A. Owens, Christopher D. Dunn, Joshua Rukundo, Rebeca Atencia, Megan F. Cole, Averill Cantwell, Melissa Emery Thompson, Alexandra G. Rosati, Tony L. Goldberg

**Affiliations:** ^1^ Department of Pathobiological Sciences, School of Veterinary Medicine University of Wisconsin‐Madison Madison Wisconsin USA; ^2^ Ngamba Island Chimpanzee Sanctuary/Chimpanzee Trust Entebbe Uganda; ^3^ Jane Goodall Institute Congo Pointe‐Noire Republic of Congo; ^4^ Department of Anthropology University of New Mexico Albuquerque New Mexico USA; ^5^ Department of Psychology University of Michigan Ann Arbor Michigan USA; ^6^ Department of Anthropology University of Michigan Ann Arbor Michigan USA

**Keywords:** conservation, health, great ape, virome, metagenomics

## Abstract

Infectious disease is a major concern for both wild and captive primate populations. Primate sanctuaries in Africa provide critical protection to thousands of wild‐born, orphan primates confiscated from the bushmeat and pet trades. However, uncertainty about the infectious agents these individuals potentially harbor has important implications for their individual care and long‐term conservation strategies. We used metagenomic next‐generation sequencing to identify viruses in blood samples from chimpanzees (*Pan troglodytes*) in three sanctuaries in West, Central, and East Africa. Our goal was to evaluate whether viruses of human origin or other “atypical” or unknown viruses might infect these chimpanzees. We identified viruses from eight families: *Anelloviridae, Flaviviridae, Genomoviridae, Hepadnaviridae, Parvoviridae, Picobirnaviridae, Picornaviridae, and Rhabdoviridae*. The majority (15/26) of viruses identified were members of the family *Anelloviridae* and represent the genera *Alphatorquevirus* (torque teno viruses) and *Betatorquevirus* (torque teno mini viruses), which are common in chimpanzees and apathogenic. Of the remaining 11 viruses, 9 were typical constituents of the chimpanzee virome that have been identified in previous studies and are also thought to be apathogenic. One virus, a novel tibrovirus (*Rhabdoviridae: Tibrovirus*) is related to Bas‐Congo virus, which was originally thought to be a human pathogen but is currently thought to be apathogenic, incidental, and vector‐borne. The only virus associated with disease was rhinovirus C (*Picornaviridae: Enterovirus*) infecting one chimpanzee subsequent to an outbreak of respiratory illness at that sanctuary. Our results suggest that the blood‐borne virome of African sanctuary chimpanzees does not differ appreciably from that of their wild counterparts, and that persistent infection with exogenous viruses may be less common than often assumed.

## Highlights

- Chimpanzee blood samples from sanctuaries in West, Central, and East Africa contained 26 viruses from eight viral families.
- Only one virus, rhinovirus C (*Picornaviridae*), has been associated with disease in captive or wild chimpanzees.
- Persistent infections with exotic or pathogenic blood‐borne viruses appear to be rare in sanctuary chimpanzees, suggesting that longstanding concerns over such viruses may be overstated.

## Graphical abstract

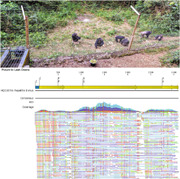

**Abbreviations**
BASV: Bas‐Congo virus
EKV‐1/2: Ekpoma virus 1 and 2
NHP: nonhuman primate
NICS: Ngamba Island Chimpanzee Sanctuary
PASA: Pan African Sanctuary Alliance
SFV: simian foamy virus
TCRC: Tchimpounga Chimpanzee Rehabilitation Centre
TCS: Tacugama Chimpanzee Sanctuary
TTMV: torque teno mini virus
TTV: torque teno virus
vRPM/kb: viral reads per million per kilobase of target sequence

## 1 INTRODUCTION

Approximately 75% of nonhuman primates (NHPs) are experiencing population declines in the wild (Estrada et al., 2017). Habitat loss, hunting, the pet trade, climate change, and disease have been implicated as causes (Gilardi et al., 2015; Humle et al., 2016; IUCN, 2021; Köndgen et al., 2008; Zhang et al., 2019). Viruses of human origin are important causes of episodic morbidity and mortality in captive and wild great ape populations (Dunay et al., 2018; Patrono et al., 2018; Scully et al., 2018). In Africa, most efforts to investigate this problem have focused on wild apes. For example, frequent respiratory disease outbreaks in wild African apes have been linked to human respiratory viruses (e.g., human metapneumovirus, human respiratory syncytial virus) (Emery Thompson et al., 2018; Grützmacher et al., 2018; Mazet et al., 2020; Negrey et al., 2019). However, collecting clinical samples from wild apes is challenging and postmortem samples are rare, such that noninvasive methods have become widely used (Grützmacher et al., 2016; Köndgen et al., 2010, 2011, 2017; Medkour et al., 2021; Negrey et al., 2020, 2022) which can limit some of these approaches.

Due to the same factors that have led to wild great ape population declines, the number of apes in African sanctuaries has increased in recent decades, as has the number of sanctuaries (Farmer, 2002; Faust et al., 2011; Ferrie et al., 2014; Hicks et al., 2010; Schoene & Brend, 2002). The Pan African Sanctuary Alliance (PASA), formed in 2000, currently has accredited 23 primate sanctuaries located in 13 countries throughout Africa that provide high‐quality care and housing for wild‐born, orphaned NHPs who have been rescued from the bushmeat and pet trades. Currently, this includes more than 1100 chimpanzees (PASA, 2021; Stokes et al., 2018), yet the fate of Africa's sanctuary apes is unclear. Sanctuaries often come under financial duress during socioeconomic crises (PASA, 2021; Stokes et al., 2018), and the number of orphans arriving at most sanctuaries continues to increase (IUCN SSC Primate Specialist Group, 2020; PASA, 2020, 2021). Strategies such as expansion, translocation, and reintroduction have all been considered to address this urgent issue but can present many logistical and ethical challenges (Beck et al., 2007; Brando et al., 2020; Humle et al., 2011; PASA, 2021; Tutin et al., 2001). A major consideration in these debates is infectious disease (Brando et al., 2020; Schaumburg et al., 2012; Sherman et al., 2021). In particular, an oft‐cited barrier to the management of sanctuary apes is the possibility that they may have acquired pathogens from humans or other “exogenous” sources. Risks for the acquisition of such viruses are high during the events surrounding capture, during the period between capture and confiscation, and during acclimatization to life at sanctuaries, due to stress, immunocompromise, and frequent close contact with people (Gilardi et al., 2014, 2015; Mugisha, Kücherer, et al., 2011; Schaumburg et al., 2012, 2013; Tutin et al., 2001).

Apes in African sanctuaries are vulnerable to many of the same infectious diseases as their wild counterparts. Previous studies using blood and/or fecal samples from sanctuary chimpanzees and utilizing traditional methods to screen for specific viral pathogens of interest are few (Ross & Leinwand, 2020). These studies have selectively focused on detecting microbes known to be transmitted between humans and apes and underlie current guidelines for sanctuary ape health assessments (PASA, 2016; Unwin et al., 2009). These microbes include the retroviruses simian foamy virus (SFV), simian immunodeficiency virus (SIV), simian T‐lymphotropic virus (STLV) (Calattini et al., 2006; Mugisha, Kücherer, et al., 2010), anelloviruses (Thom et al., 2003), herpesviruses (Leendertz et al., 2009; Mugisha, Leendertz, et al., 2010, Mugisha, Kücherer, et al., 2011), hepatitis viruses (B, C, and E) (Lyons et al., 2012; MacDonald et al., 2000; Mugisha, Kaiser, et al., 2011, Mugisha, Kücherer, et al., 2011; Starkman et al., 2003), flaviviruses, human metapneumovirus, and chikungunya virus (Mugisha, Kücherer, et al., 2011), polyomaviruses (Scuda et al., 2013), enteroviruses (Sadeuh‐Mba et al., 2014), and adenoviruses (Mugisha, Kücherer, et al., 2011; Wevers et al., 2011). Next‐generation DNA sequencing, which allows for unbiased, broad detection of viruses, has, to our knowledge, been reported only in a single instance: a case of acute flaccid paralysis in a sanctuary chimpanzee in Republic of Congo, revealing a human enterovirus C (*Picornaviridae*) strain (Mombo et al., 2015, 2020).

Sanctuary apes also provide an intriguing system for studying the “human‐wildlife interface,” albeit in a context different from how it is typically depicted (Devaux et al., 2019; Namusisi et al., 2021). Unlike their conspecifics in many captive facilities outside of Africa, African sanctuary apes typically semi‐free‐range in enclosures containing forested habitat and live in social groups similar in size and composition to those of wild apes (PASA, 2016). While the nutritional status of sanctuary apes is far more stable than that of wild apes, most sanctuaries provide a diet that mimics that of wild apes, and apes can also forage within their enclosures (PASA, 2016). In this way, African sanctuary populations show patterns of physiological health and behavior that better mirrors wild populations than do captive populations outside of Africa (Cole et al., 2020; Rosati et al., 2013; Wobber & Hare, 2011). Sanctuary apes also come into direct contact or close proximity to human caretakers daily, and tourists visit many ape sanctuaries as well, providing opportunities for pathogen transmission in both directions (Gilardi et al., 2014; Glasser et al., 2021; Macfie & Williamson, 2010; Schaumburg et al., 2012). Moreover, blood sampling is possible during routine health checks, usually conducted annually (PASA, 2016; Unwin et al., 2009), allowing for analyses that are not feasible in wild chimpanzee populations.

In this study, we used metagenomic next‐generation sequencing to characterize and compare the blood viromes of three populations of sanctuary chimpanzees in Equatorial Africa: (A) Tacugama Chimpanzee Sanctuary (TCS) in Sierra Leone, (B) Tchimpounga Chimpanzee Rehabilitation Centre (TCRC) in Republic of Congo, and (C) Ngamba Island Chimpanzee Sanctuary (NICS) in Uganda. To our knowledge, this is the first study to use such methods to characterize the viromes of sanctuary‐housed chimpanzees. This work complements studies of pathogens in wild‐living apes, where such blood samples cannot routinely be obtained. The study also addresses the possibility that apes in sanctuaries harbor “unusual” viruses from exogenous sources, which could impact management options and conservation strategies.

## 2 METHODS

### 2.1 Study sites

**Figure 1:**
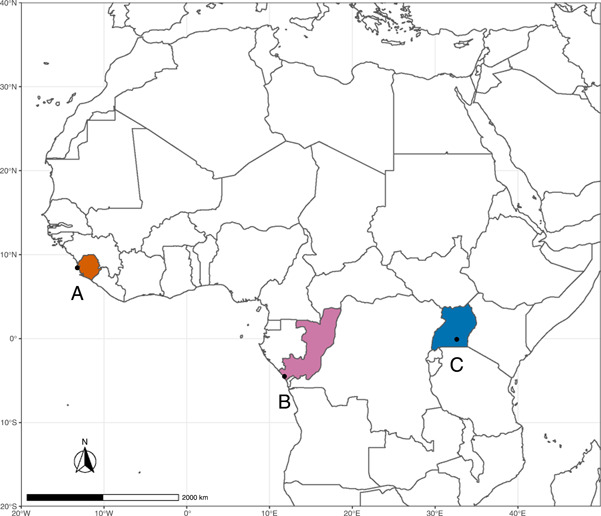
(A) Tacugama Chimpanzee Sanctuary (TCS) in Sierra Leone (orange). (B) Tchimpounga Chimpanzee Rehabilitation Centre (TCRC) in Republic of Congo (purple). (C) Ngamba Island Chimpanzee Sanctuary (NICS) in Uganda (blue). Map created using R v. 4.0.2 (R Core Team, 2020).

The study sites included three PASA member chimpanzee sanctuaries: (A) TCS, (B) TCRC, and (C) NICS (Figure 1). TCS, founded in 1995, is located in Western Area National Park, Freetown, Sierra Leone. TCS cares for approximately 100 chimpanzees. TCRC is located north of Pointe‐Noire, Republic of Congo, within the larger Tchimpounga Nature Reserve. TCRC was established in 1992 and cares for approximately 150 chimpanzees. NICS, founded in 1998, occupies Ngamba Island, part of the Koome Island group in Lake Victoria, Uganda, and cares for approximately 52 chimpanzees. Chimpanzees at all three sanctuaries semi‐free‐range in forested enclosures and live in species‐typical social groups with continuous full‐group contact (i.e., housed together with unimpeded physical contact). Except for instances of contraceptive failure, TCS, TCRC, and NICS resident chimpanzees are wild‐born individuals who have been rescued from the illegal bushmeat and pet trades as infants or juveniles.

### 2.2 Study populations and sample collection

All blood sample collection occurred during routine health examinations performed by sanctuary veterinarians for the care and welfare of the chimpanzees. For TCS, we used published data from samples collected between March 15, 2013, and July 11, 2016, as previously described (Owens et al., 2021). Samples from TCS included blood collected during health examinations and organs collected during necropsies. We analyzed samples from 16 wild‐born chimpanzees (6 males and 10 females, ages 5–21 years old) (Supporting Information: Table S1). At TCRC, we collected samples between June 16 and June 20, 2019, from 1 sanctuary‐born and 24 wild‐born chimpanzees (17 males and 8 females, ages 14–23 years old) (Supporting Information: Table S1), as previously described (Cole et al., 2020). Chimpanzees were fully anesthetized, and blood was collected via venipuncture from either the femoral vein or median cubital vein into BD Vacutainer PPT plasma preparation tubes (Becton, Dickinson and Company, Inc.). We centrifuged PPT tubes at 350 RPM for 10 min. Because of the unavailability of liquid nitrogen, TCRC plasma samples were mixed with 2× DNA/RNA Shield (Zymo Research) in a 1:1 ratio before storage at −20°C, to stabilize nucleic acids. At NICS, we collected blood samples between July 18 and August 20, 2016, from 28 wild‐born chimpanzees (12 males and 16 females, ages 7–32 years old) (Supporting Information: Table S1), using the same methods described for TCRC. NICS plasma samples were frozen in liquid nitrogen. Samples were kept frozen during shipment to the USA and were stored at −80°C until processing (i.e., the “cold chain” was unbroken).

### 2.3 Sample preparation and next‐generation sequencing

We performed metagenomic/metatranscriptomic next‐generation sequencing as previously described (Owens et al., 2021). Briefly, we isolated viral nucleic acids from plasma using the QIAmp MinElute Virus Spin Kit (Qiagen), and converted viral RNA to double‐stranded complementary DNA (cDNA) using the SuperScript double‐stranded cDNA Synthesis Kit (Invitrogen) which was then purified using AmpureXP beads (Beckman Coulter). We prepared libraries using the Nextera XT DNA sample preparation kit (Illumina) for sequencing on a MiSeq instrument (MiSeq Reagent Kit, V2 chemistry, 300 cycles; Illumina). We have used these methods previously to identify diverse viruses in NHP samples (e.g., Bennett et al., 2016; Kuhn, Sibley, et al., 2020; Ladner et al., 2016; Lauck et al., 2011; Lauck, Sibley, Hyeroba, et al., 2013; Lauck, Sibley, Lara, et al., 2013; Lauck, Switzer, et al., 2013; Lauck et al., 2014; Negrey et al., 2019, 2020, 2022; Scully et al., 2018; Sibley et al., 2014).

### 2.4 Bioinformatic analysis

We analyzed sequence data using CLC Genomics Workbench v. 20.0.4 (Qiagen) as previously described (Bennett et al., 2020; Negrey et al., 2020, 2022; Owens et al., 2021; Ramírez‐Martínez et al., 2021). Briefly, we trimmed sequences of low quality (Phred quality score <30) and short length (<50 bp) and removed sequences matching host DNA and known contaminants. We subjected remaining reads to de novo assembly and compared resulting contiguous sequences to the GenBank database using both the BLASTn (nucleotide‐level) and BLASTx (protein‐level) algorithms (Altschul et al., 1990; Gish & States, 1993). We removed viruses not associated with mammalian hosts (e.g., bacteriophages) from further consideration.

To identify positive individuals and to quantify intensities of infection, we mapped reads from each individual to the sequence of a conserved target gene (e.g., polymerase) of each virus identified (length fraction = 1.0; similarity = 0.9). We considered an individual positive if ≥1 read of ≥50 bases matched a virus in the database (Negrey et al., 2020, 2022; Ramírez‐Martínez et al., 2021). We estimated viral load by calculating the proportion of reads mapping to each virus (viral load) and the proportion of reads mapping to any virus in the population (total viral load). We then normalized this measure to one million reads and to the length of the target sequence for each virus and applied a log transformation (log_10_ viral reads per million per kilobase of target sequence, or log_10_vRPM/kb), which is a metric correlated with real‐time quantitative polymerase chain reaction data (Toohey‐Kurth et al., 2017) and has been previously used to quantify viruses in chimpanzees (Negrey et al., 2020, 2022).

### 2.5 Phylogenetic analyses

To infer phylogenetic relationships among viruses, we used the codon‐guided Prank algorithm (Löytynoja, 2014), implemented by TranslatorX (Abascal et al., 2010), to align published nucleotide sequences of related viruses in the GenBank database and our newly identified viruses, and we removed poorly aligned regions using the Gblocks algorithm (Castresana, 2000). Using the resulting nucleotide alignments, we generated maximum‐likelihood phylogenetic trees using PhyML 3.0 with 1000 bootstrap replicates (Guindon et al., 2010; Lefort et al., 2017). We displayed final trees using FigTree v. 1.4.4 (Rambaut, 2018). We determined percentage identity shared between sequences using Clustal Omega (Madeira et al., 2019). We calculated within‐ and between‐sanctuary population genetic distances (nucleotide p‐distance and standard error calculated with 1000 bootstrap replicates) using MEGA X (Kumar et al., 2018).

### 2.6 Statistical analyses

We calculated the prevalence of each virus as the proportion of positive individuals, with 95% confidence intervals using the modified Wald method (Agresti & Coull, 1998). We assessed the association between sex (male or female) and viral prevalence using odds ratios with 95% confidence intervals and two‐tailed Fisher's exact tests (“fisher.test” in R) and the association between sex and viral load (infected individuals only) using Mann–Whitney *U*/Wilcoxon rank‐sum tests (“wilcox.test”). We compared viral richness (number of viruses per individual) and total viral load among the three sanctuaries using Kruskal–Wallis tests (“kruskal.test”) and subsequent Wilcoxon rank‐sum tests with the Benjamini‐Hochberg adjustment to control for multiple comparisons (“pairwise.wilcox.test” with p.adjust.method = “BH”) (Benjamini & Hochberg, 1995). We conducted statistical analyses using R v. 4.0.2 (R Core Team, 2020).

## 3 RESULTS

### 3.1 Characterization of viruses

**Figure 2:**
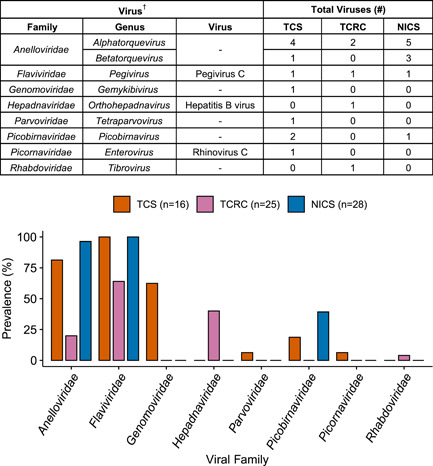
Viral prevalence in sanctuary chimpanzees at TCS, TCRC, and NICS. Barplot displays the proportion of individuals at each sanctuary who were positive for at least one virus from the family. ^†^Viral genera were assigned using phylogenetic analyses (Supporting Information: Figures S1‐S2). NICS, Ngamba Island Chimpanzee Sanctuary; TCRC, Tchimpounga Chimpanzee Rehabilitation Centre; TCS, Tacugama Chimpanzee Sanctuary.

We generated an average of 3,757,384 reads per sample (SD ± 2,716,857) for TCS, 1,671,859 reads per sample (SD ± 475,745) for TCRC, and 2,357,429 reads per sample (SD ± 281,042) for NICS after quality and length trimming. In total, we identified 26 viruses (11 at TCS, 5 at TCRC, and 10 at NICS) of five genome types (ssRNA[+], ssRNA[−], dsRNA, ssDNA, and dsDNA‐RT) (Table 1). Nucleotide sequence identity to known viruses ranged from 65.84% to 100%. We further identified the viruses as representing eight viral families: *Anelloviridae, Flaviviridae, Genomoviridae, Hepadnaviridae, Parvoviridae, Picobirnaviridae*, *Picornaviridae*, and *Rhabdoviridae* (Figure 2 and Supporting Information: Figures S1, S2). We identified members of two virus taxa at all three sanctuaries: anelloviruses (*Anellovriidae*), classified as either a torque teno viruses (TTVs: genus *Alphatorquevirus*) or torque teno mini viruses (TTMVs: genus *Betatorquevirus*), and pegiviruses (*Flaviviridae*), previously referred to as “GB virus C” (Stapleton et al., 2011). At TCS and NICS only, we identified picobirnaviruses (*Picobirnaviridae*). We found members of the remaining five virus taxa in chimpanzees from only a single sanctuary: gemykivibivirus (*Genomoviridae*), tetraparvovirus (*Parvoviridae*), and rhinovirus C (*Picornaviridae*) at TCS, and hepatitis B virus (*Hepadnaviridae*) and tibrovirus (*Rhabdoviridae*) at TCRC.

**Table 1:** Viruses identified in sanctuary chimpanzees

ID	Virus	Sanctuary	Abbreviation	Genome	Closest match (source, location, year, accession)^a^	Family^a^	Genus^a^	E‐Value^a^	% ID (NT)^a^	Accession^b^
1	Chimpanzee GB virus C	TCS	TAPTV‐8	ssRNA+	GB virus C variant troglodytes (chimpanzee, USA, 1998, AF070476)	*Flaviviridae*	*Pegivirus*	0	90.33	MT350348
2	Ticpantry virus 5	TCRC	TCPTV‐5	ssRNA+	GB virus C variant troglodytes (chimpanzee, USA, 1998, AF070476)	*Flaviviridae*	*Pegivirus*	0	75.75	ON706347
3	Nabpantry virus 9	NICS	NAPTV‐9	ssRNA+	Chimpanzee GB virus C (chimpanzee, Sierra Leone, 2013‐2016, MT350348)	*Flaviviridae*	*Pegivirus*	0	78.13	ON706343
4	Chimpanzee rhinovirus C	TCS	TAPTV‐9	ssRNA+	Rhinovirus C (human, USA, 2015, MG148341)	*Picornaviridae*	*Enterovirus*	0	95.83	MT350353
5	Ticpantry virus 4	TCRC	TCPTV‐4	ssRNA‐	Ekpoma virus (human, China, 2017, MF079256)	*Rhabdoviridae*	*Tibrovirus*	4.00E−101	65.84	ON706348
6	Chimpanzee picobirnavirus isolate chimpanzee1	TCS	TAPTV‐10	dsRNA (linear)	Human picobirnavirus (human, USA, 1991, AF246940)	*Picobirnaviridae*	*Picobirnavirus*	0	99.1	MT350351
7	Chimpanzee picobirnavirus isolate chimpanzee2	TCS	TAPTV‐11	dsRNA (linear)	Macaque picobirnavirus 21 (macaque, USA, 2011, MG010906)	*Picobirnaviridae*	unclassified	0	78.7	MT350352
8	Nabpantry virus 10	NICS	NAPTV‐10	dsRNA (linear)	Porcine picobirnavirus (pig, Italy, 2004, KF861773)	*Picobirnaviridae*	unclassified	0	77.36	ON706344
9	Chimpanzee anellovirus	TCS	TAPTV‐1	ssDNA (circular)	Chimpanzee anellovirus (chimpanzee, Czech Republic, 2012, KT027937)	*Anelloviridae*	unclassified	0	87.02	MT350347
10	Chimpanzee torque teno virus isolate chimpanzee1	TCS	TAPTV‐2	ssDNA (circular)	Torque teno virus (human, Tanzania, 2015, MN767291)	*Anelloviridae*	unclassified	1.00E−126	74.07	MT350354
11	Chimpanzee torque teno virus isolate chimpanzee2	TCS	TAPTV‐3	ssDNA (circular)	Torque teno virus (human, Tanzania, 2015, MN767387)	*Anelloviridae*	unclassified	0	75.33	MT350355
12	Chimpanzee torque teno virus isolate chimpanzee3	TCS	TAPTV‐4	ssDNA (circular)	Torque teno virus 14 (chimpanzee, West Africa, 2000, AB037926)	*Anelloviridae*	*Alphatorquevirus*	0	88.25	MT350356
13	Chimpanzee torque teno virus isolate chimpanzee4	TCS	TAPTV‐5	ssDNA (circular)	Torque teno virus 23 (chimpanzee, Japan, 2000, NC_038342)	*Anelloviridae*	*Alphatorquevirus*	0	90.7	MT350357
14	Ticpantry virus 1	TCRC	TCPTV‐1	ssDNA (circular)	Torque teno virus (human, Tanzania, 2015, MN767404)	*Anelloviridae*	unclassified	5.00E−46	77.04	ON706345
15	Ticpantry virus 2	TCRC	TCPTV‐2	ssDNA (circular)	Chimpanzee torque teno virus (chimpanzee, Sierra Leone, 2013‐2016, MT350354)	*Anelloviridae*	unclassified	2.00E−177	84.56	ON706346
16	Nabpantry virus 1	NICS	NAPTV‐1	ssDNA (circular)	Gorilla anellovirus (gorilla, Czech Republic, 2012, KT027941)	*Anelloviridae*	*Omegatorquevirus*	9.00E−56	69.64	ON706335
17	Nabpantry virus 2	NICS	NAPTV‐2	ssDNA (circular)	Chimpanzee anellovirus (chimpanzee, Sierra Leone, 2013‐2016, MT350347)	*Anelloviridae*	unclassified	0	73.49	ON706336
18	Nabpantry virus 3	NICS	NAPTV‐3	ssDNA (circular)	Torque teno virus (human, Tanzania, 2015, MN767395)	*Anelloviridae*	*Alphatorquevirus*	0	82.2	ON706337
19	Nabpantry virus 4	NICS	NAPTV‐4	ssDNA (circular)	TTV‐like mini virus (human, Tanzania, 2015, MN773698)	*Anelloviridae*	*Betatorquevirus*	0	73.65	ON706338
20	Nabpantry virus 5	NICS	NAPTV‐5	ssDNA (circular)	Torque teno virus 23 (chimpanzee, Japan, 2000, NC_038342)	*Anelloviridae*	*Alphatorquevirus*	0	70.46	ON706339
21	Nabpantry virus 6	NICS	NAPTV‐6	ssDNA (circular)	Torque teno virus (human, Tanzania, 2015, MN767891)	*Anelloviridae*	unclassified	2.00E−109	69.52	ON706340
22	Nabpantry virus 7	NICS	NAPTV‐7	ssDNA (circular)	Torque teno virus 14 (chimpanzee, Japan, 2000, AB037926)	*Anelloviridae*	*Alphatorquevirus*	0	78.06	ON706341
23	Nabpantry virus 8	NICS	NAPTV‐8	ssDNA (circular)	Torque teno virus (human, Tanzania, 2015, MN767542)	*Anelloviridae*	unclassified	0	72.46	ON706342
24	Chimpanzee gemykibivirus	TCS	TAPTV‐6	ssDNA (circular)	Gemycircularvirus NP (sewage, Nepal, 2009, KP133080)	*Genomoviridae*	*Gemykibivirus*	0	100	MT350349
25	Chimpanzee parvovirus	TCS	TAPTV‐7	ssDNA (linear)	Parvovirus 4‐like MK‐2012 (chimpanzee, Cote d'Ivoire, 2002, JN798204)	*Parvoviridae*	*Protoparvovirus*	2.00E−105	98.25	MT350350
26	Ticpantry virus 3	TCRC	TCPTV‐3	dsDNA‐RT (circular)	Hepatitis B virus (chimpanzee, Uganda, 2001, HQ018764)	*Hepadnaviridae*	*Orthohepadnavirus*	0	95.52	ON706349

^a^ Closest match, family, genus, E‐value, and percent identity (nucleotide) were identified by querying the polymerase (ssRNA‐, dsRNA, and dsDNA‐RT viruses), genomic polyprotein (ssRNA + viruses), or replication‐associated protein (ssDNA viruses) nucleotide sequence against the NCBI's nonredundant nucleotide database using the discontiguous megablast homology searching algorithm.

^b^ Accession number of viral sequence from this study and as previously reported for TCS in Owens et al. (2021).

Anelloviruses were the majority of viruses identified (15/26; 57.7%). The partial or complete ORF1 sequences of the TTVs shared 48% amino acid (aa) and 52% nucleotide (nt) identity (ID) with each other, on average. The TTMVs shared 45% aa and 57% nt ID with each other, on average. For the TTVs identified in each sanctuary population, within‐population genetic distances (0.56 ± 0.01 to 0.77 ± 0.05) were similar to between‐population genetic distances (0.56 ± 0.01 to 0.64 ± 0.01). Similarly, for the TTMVs identified at TCS and NICS, within‐population genetic distance (0.45 ± 0.01; NICS only) was similar to between‐population genetic distance (0.40 ± 0.01). The complete polyprotein sequences of the three pegiviruses (TAPTV‐8, TCPTV‐5, and NAPTV‐9) shared ~88% aa and ~76% nt ID with each other. The three picobirnaviruses were classified into two genogroups (genogroup 1: TAPTV‐10 and NAPTV‐10; genogroup 2: TAPTV‐11) (Figure S1). Within genogroup 1, complete or partial RNA‐dependent RNA polymerase sequences shared 68% aa and 64% nt ID. The two picobirnavirus genogroups shared ~26% aa and ~41% nt ID. For the remaining viral families, *Genomoviridae, Hepadnaviridae, Parvoviridae, Picornaviridae*, and *Rhabdoviridae*, we identified only one virus, obviating the need for such comparisons.

The TTVs shared, on average, 43% aa and 54% nt ID with select human TTVs (GenBank accessions: KT163901, MW455400, AB064607, AB028668). The TTMVs shared, on average, 43% aa and 61% nt ID with select human TTMVs (GenBank accessions: AB038630, EF538880, JX134046). The complete polyprotein sequences of the three pegiviruses (TAPTV‐8, TCPTV‐5, and NAPTV‐9) shared ~80% aa and ~71% nt ID with human pegivirus C genotypes 1, 2, and 5, which have been detected in Africa (GenBank accessions: U63715, U44402, AY949771; Singh & Blackard, 2017). The partial NS1 sequence of the tetraparvovirus identified at TCS (TAPTV‐7) shared ~88% aa ~79% nt ID with human tetraparvovirus (PARV4) which is endemic in Africa (GenBank accessions: AY622943, DQ873390, EU874248; Cotmore et al., 2019). The complete polymerase sequence of the hepatitis B virus (TCPTV‐3) identified at TCRC shared ~86% aa and ~91% nt ID with human hepatitis B virus genotypes A and E, which have been detected in the Republic of Congo (GenBank accessions: X02763 and X75657; Ghoma Linguissi & Nkenfou, 2017).

### 3.2 Viral prevalence, load, and richness

At least one anellovirus was detected in 20% (95% confidence interval [CI]: 8.4%, 39.6%) to 96.4% (95% CI: 80.8%, >99.9%) of chimpanzees at each sanctuary (Figure 2). Pegivirus C was the most prevalent virus at each sanctuary, infecting 64% (95% CI: 44.4%, 79.8%) of individuals at TCRC and 100% of individuals at TCS (95% CI: 77.3%, 100%) and NICS (95% CI: 85.7%, 100%). A picobirnavirus was identified in 18.8% (95% CI: 5.8%, 43.8%) of TCS individuals and 39.3% (95% CI: 23.5%, 57.6%) of NICS individuals. The gemykibivirus was present in 62.5% (95% CI: 38.5%, 81.6%) of TCS individuals and the hepatitis B virus was present in 40% (95% CI: 23.4%, 59.3%) of TCRC individuals. The least prevalent viruses overall, each found in only one individual, were the tetraparvovirus and rhinovirus C, both 6.3% (95% CI: < 0.01%, 30.3%) at TCS, and the tibrovirus, 4% (95% CI: < 0.01%, 21.1%) at TCRC. Prevalence values of each virus overall and stratified by the sex of the chimpanzee are provided in Supporting Information: Table S2. There were no statistically significant associations between sex and viral prevalence.

**Figure 3:**
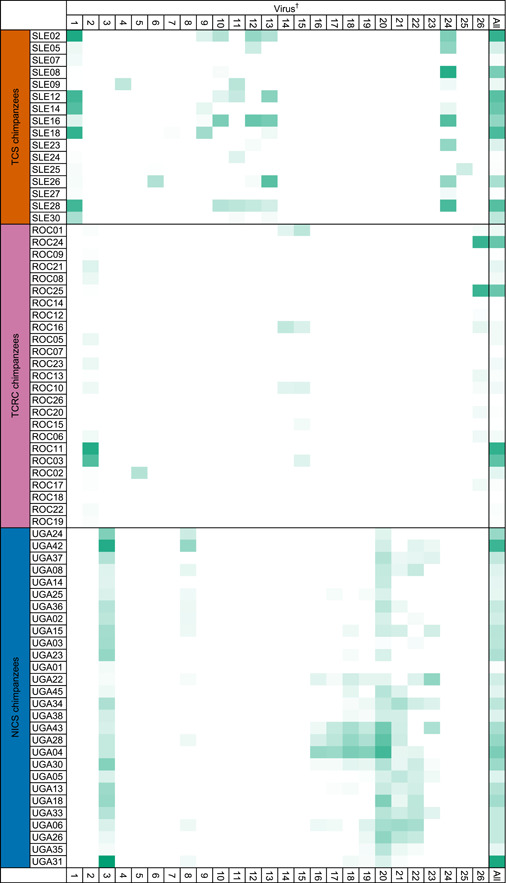
Heatmap of viral loads of sanctuary chimpanzees at TCS, TCRC, and NICS. Displays viral load data (log_10_vRPM/kb for each virus: 1–26) and total viral load data (log_10_vRPM/kb for all viruses) for each individual at each sanctuary. Values range from 0 (lightest) to 4.0 (darkest). ^†^Refers to ID in Table 1. NICS, Ngamba Island Chimpanzee Sanctuary; TCRC, Tchimpounga Chimpanzee Rehabilitation Centre; TCS, Tacugama Chimpanzee Sanctuary.

**Figure 4:**
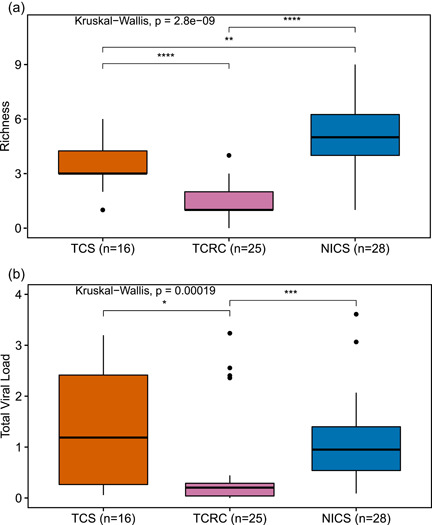
Boxplots of (a) viral richness and (b) total viral load (log_10_vRPM/kb) for each sanctuary chimpanzee population (TCS, TCRC, and NICS). Kruskal–Wallis test significant *p* values (<0.05) are displayed. Significant *p* values for subsequent pairwise comparisons using Wilcoxon rank‐sum tests with the Benjamini‐Hochberg adjustment applied are indicated as follows: *p < 0.05, **p < 0.01, ***p < 0.001, ****p < 0.0001. Original and adjusted *p* values are provided in Supporting Information: Table S6. NICS, Ngamba Island Chimpanzee Sanctuary; TCRC, Tchimpounga Chimpanzee Rehabilitation Centre; TCS, Tacugama Chimpanzee Sanctuary.

Viral load varied from 0 to 4 log_10_vRPM/kb overall (TCS: 0–3.55, TCRC: 0–3.44, NICS: 0‐4) (Figure 3 and Supporting Information: Tables S3‐S5). Total viral load ranged from 0 to 3.61 log_10_vRPM/kb overall (TCS: 0.05–3.2, TCRC: 0–3.24, NICS: 0.09–3.61). The mean total viral load was 1.36 (SD ± 1.14) at TCS, 0.64 (SD ± 0.95) at TCRC, and 1.13 (SD ± 0.78) at NICS. The gemykibivirus (TAPTV‐6) had the highest mean viral load among infected individuals (1.7 log_10_vRPM/kb). A picobirnavirus (TAPTV‐11) had the lowest mean viral load among infected individuals (0.09 log_10_vRPM/kb). We compared the distribution of total viral load for the three sanctuaries and found a statistically significant difference (Kruskal–Wallis *p* = 0.00019) (Figure 4). Subsequent pairwise comparisons using Wilcoxon rank‐sum tests showed a difference in total viral load for two pairs (TCS:TCRC *p* = 0.011; NICS:TCRC *p* = 0.00013; Benjamini‐Hochberg adjustment) (Supporting Information: Table S6). There were no statistically significant differences in viral load between male and female chimpanzees (Supporting Information: Table S2).

Viral richness varied from 0 to 9 overall (TCS: 1–6, TCRC: 0–4 NICS: 1–9) (Supporting Information: Tables S3‐S5). Mean viral richness was 3.6 (SD ± 1.5) at TCS, 1.4 (SD ± 1.1) at TCRC, and 5.3 (SD ± 2.1) at NICS. TCRC was the only sanctuary in which some individuals were not infected with any virus (1 female age 19 years old and 3 males ages 16.5–22 years old). We compared the distribution of viral richness among the three sanctuaries and found a statistically significant difference (Kruskal–Wallis *p* = 2.8e−09) (Figure 4). Subsequent pairwise comparisons using Wilcoxon rank‐sum tests showed a significant difference in viral richness for each pair (TCS:NICS *p* = 7.6e−03; TCS:TCRC *p* = 3.53e−05; NICS:TCRC *p* = 3.3e−08; Benjamini–Hochberg adjustment) (Supporting Information: Table S6).

## 4 DISCUSSION

In this study, we characterized the blood virome of three African sanctuary chimpanzee populations and found them to be infected with 26 viruses from eight families at prevalences ranging from 4% to 100%. We observed significant differences in viral richness and total viral load among the three sanctuary populations during the study period. Specifically, chimpanzees at TCRC exhibited lower viral richness and lower total viral load than chimpanzees at either TCS or NICS. This observation could be a result of differences in various factors such as chimpanzee geographic origin, history before arrival at the sanctuary, or social group dynamics. With two exceptions (rhinovirus C and the novel tibrovirus; see below), the viruses we identified in these populations are “normal” members of the chimpanzee virome, in that they are known to infect chimpanzees without clinical consequences.

Specifically, healthy captive and wild chimpanzees are known to be infected with anelloviruses (Abe et al., 2000; Hrazdilová et al., 2016; Thom et al., 2003), tetraparvoviruses (Adlhoch et al., 2012; Cotmore et al., 2019; Sharp et al., 2010), pegiviruses (Lewis et al., 1998; Mohr et al., 2011; Simmonds et al., 2017; Stapleton et al., 2011), and chimpanzee‐specific hepatitis B viruses (Hu et al., 2000; Lyons et al., 2012; MacDonald et al., 2000; Makuwa et al., 2005; Mugisha, Kaiser, et al., 2011; Starkman et al., 2003; Vartanian et al., 2002). These viruses are considered commensal and apathogenic in chimpanzees. Each of these viruses is present in the global human population, but only hepatitis B virus is considered to be pathogenic (Cotmore et al., 2019; Hrazdilová et al., 2016; Lyons et al., 2012; Simmonds et al., 2017). Human and chimpanzee anelloviruses do not form distinct clusters phylogenetically (Supporting Information: Figure S1; Fahsbender et al., 2017; Hrazdilová et al., 2016; Kaczorowska & van der Hoek, 2020; Okamoto et al., 2000). In contrast, chimpanzee tetraparvoviruses, pegiviruses, and hepatitis B viruses each cluster together separately from human viruses (Supporting Information: Figures S1‐S2; Adlhoch et al., 2012; Locarnini et al., 2021; Sibley et al., 2014). Transmission of tetraparvoviruses between humans and chimpanzees has not been observed (Adlhoch et al., 2012). Chimpanzees have been experimentally infected with human anelloviruses, human pegivirus C, and human hepatitis B virus, with hepatitis B virus causing disease in chimpanzees; however, evidence for natural cross‐species transmission is lacking (Simmonds et al., 2017; Tawara et al., 2000; Wieland, 2015). Hepatitis B virus testing and vaccination for humans and chimpanzees is advised for PASA sanctuary management to mitigate the risk of zoonotic transmission (Hu et al., 2000; PASA, 2016).

Picobirnaviruses (Delmas et al., 2019; Duraisamy et al., 2018; Ghosh & Malik, 2021; Malik et al., 2014; Negrey et al., 2020) and gemykibiviruses (Varsani & Krupovic, 2021; J. Wang et al., 2019) have been widely detected in vertebrates, invertebrates, and environmental sources. The true hosts of these viruses are unknown (they may actually be viruses of fungi; Ghosh & Malik, 2021; D. Wang, 2022; J. Wang et al., 2019), and they have not been associated with disease.

To our knowledge, this study is the first to identify a tibrovirus in any NHP, ticpantry virus 4, in a 19‐year‐old female chimpanzee at TCRC. The genus *Tibrovirus* is poorly characterized in comparison to many other members of the family *Rhabdoviridae* (Kuhn, Pān, et al., 2020). Tibroviruses have been identified in cattle, humans, and biting midges, a known vector of nonhuman tibroviruses. Bas‐Congo virus (BASV) was initially identified in human serum from a survivor of a hemorrhagic fever in Democratic Republic of Congo in 2009, leading to suspicions that it might be a frank pathogen (Grard et al., 2012). However, the related tibroviruses, Ekpoma virus 1 and 2 (EKV‐1/2), were subsequently identified in plasma from healthy people in Nigeria (Stremlau et al., 2015). Seroprevalence studies show that human tibroviruses commonly infect healthy humans in West and Central Africa (Edridge et al., 2022; Kuhn, Pān, et al., 2020). At present, pathogenicity has not been established for any tibrovirus. Ticpantry virus 4 in the plasma of a healthy sanctuary‐housed chimpanzee in Central Africa may therefore reflect an unknown diversity of tibroviruses in the region. The fact that BASV and EKV‐1/2 may be transmitted by biting midges (Babayan et al., 2018; Kuhn, Pān, et al., 2020) suggests that ticpantry virus 4 may also be vector‐borne.

The only virus identified in this study known to cause disease in chimpanzees is rhinovirus C, which we detected in one individual (a 5‐year‐old male) at TCS in 2016, coinciding with an outbreak of respiratory illness. Rhinovirus C caused a lethal outbreak of respiratory illness in the Kanyawara chimpanzees of Kibale National Park, Uganda, in 2013 (Scully et al., 2018). Human rhinoviruses are the predominant cause of upper respiratory tract infections in humans and, before the outbreak at Kibale, were known to infect only humans (Jacobs et al., 2013). Because rhinovirus C is typically detected in the respiratory tract and quickly cleared, viremia is uncommon and associated with severe infections in humans (Bochkov & Gern, 2012).

Overall, our results show that sanctuary chimpanzees are infected with commensal, apathogenic viruses, many of which have been documented in wild chimpanzees, and that reverse zoonotic transmission of respiratory viruses occurs in sanctuaries as it does in the wild. We did not find evidence of exogenous viruses in the blood of sanctuary chimpanzees. Historically, concern over the possibility of sanctuary apes being infected with “atypical” pathogens has impeded conservation and management efforts and halted discussions about reintroduction of wild‐born, sanctuary‐housed individuals to the wild. Although reintroduction is challenging and not a goal for all primate sanctuaries, it is a conservation tool that some have considered under certain circumstances (Beck et al., 2007; Humle et al., 2011). Our results suggest that concerns about persistent or prevalent infection of chimpanzees with exogenous blood‐borne viruses may be overstated.

Our study was limited to blood plasma samples collected cross‐sectionally. We expect that other sample types (e.g., feces, saliva, urine) would yield different results, due to tissue‐specific viral tropism (McFadden et al., 2009). For example, chimpanzees harbor enterotropic (i.e., gastrointestinal) viruses, such as enteroviruses (Mombo et al., 2015; Sadeuh‐Mba et al., 2014) and adenoviruses (Mugisha, Kücherer, et al., 2011; Wevers et al., 2011), cell‐associated viruses, such as foamy viruses (Calattini et al., 2006; von Laer et al., 1996; Mugisha, Kücherer, et al., 2010) and herpesviruses (Gatherer et al., 2021; Leendertz et al., 2009; Mugisha, Leendertz, et al., 2010). Absence or transient viremia may explain why we did not detect viruses such as SFV or gammaherpesviruses, which have been reported previously in chimpanzees at NICS (Mugisha, Kücherer, et al., 2010; Mugisha, Leendertz, et al., 2010). Bacterial and parasitic pathogens also are an important component of sanctuary ape health assessments and management considerations, and this study did not examine those classes of agents (Beck et al., 2007; Mugisha et al., 2014; Owens et al., 2021; Schaumburg et al., 2012). Additionally, we acknowledge that there are limitations associated with the use of metagenomics for inferring viral infection in chimpanzees (Negrey et al., 2022). For example, the sensitivity of our methods for classifying a sample as positive depends on the chosen positivity threshold (≥1 read mapping to the virus, in our case). Fortunately, exploring different positivity thresholds (both absolute and relative to sequencing depth) did not change any of our statistical inferences or conclusions (data not shown).

Captive populations of many animals are becoming increasingly important as repositories of genetic diversity, but lack of knowledge of infection status currently limits management options. Our approach, if more extensively and intensively applied, could help address this problem. For example, it could help guide the establishment of new captive populations from existing ones, with the goal of excluding particular viruses (similar to “specific pathogen‐free” laboratory animal colonies; Yee et al., 2016). Our approach could also help inform reintroduction efforts, especially for species where reintroduction is less controversial than for great apes.

## AUTHOR CONTRIBUTIONS

**Emily Dunay**: Conceptualization (equal); Formal analysis (equal); Investigation (equal); Methodology (equal); Writing – original draft (equal); Writing – review and editing (equal). **Leah A. Owens**: Formal analysis (equal); Writing – review and editing (equal). **Christopher D. Dunn**: Investigation (equal); Writing – review & editing (equal). **Joshua Rukundo**: Investigation (equal); Methodology (equal); Resources (equal); Writing – review and editing (equal). **Rebeca Atencia**: Investigation (equal); Methodology (equal); Resources (equal); Writing – review and editing (equal). **Megan F. Cole**: Investigation (equal); Writing – review and editing (equal). **Averill Cantwell**: Investigation (equal); Writing – review and editing (equal). **Melissa E. Thompson**: Conceptualization (equal); Funding acquisition (equal); Investigation (equal); Methodology (equal); Supervision (equal); Writing – review and editing (equal). **Alexandra G. Rosati**: Conceptualization (equal); Funding acquisition (equal); Investigation (equal); Methodology (equal); Resources (equal); Supervision (equal); Writing – review and editing (equal). **Tony L. Goldberg**: Conceptualization (equal); Funding acquisition (equal); Investigation (equal); Methodology (equal); Resources (equal); Supervision (equal); Writing – original draft (equal); Writing – review and editing (equal).

## CONFLICT OF INTEREST

The authors declare no conflict of interest.

## ETHICS STATEMENT

Research at TCS was approved by the Government of Sierra Leone Ministry of Agriculture, Forestry, and Food Security. Research at TCRC was approved by the Republic of Congo Ministry of Scientific Research and Technological Innovation and Jane Goodall Institute Congo. NICS is managed by Chimpanzee Sanctuary and Wildlife Conservation Trust (CSWCT) and research was approved by CSWCT, the Uganda Wildlife Authority, and the Uganda National Council for Science and Technology. Work at TCRC and NICS was approved by the Institutional Animal Care and Use Committee at the University of Michigan (#8102) and Harvard University (#14‐07‐206‐1). All samples were shipped to the USA under Convention on International Trade in Endangered Species of Wild Fauna and Flora permits (Sierra Leone permit 0000004 and USA permit 17US19807C/9 [TCS]; Republic of Congo permit CG1126038 and USA permit 20US56953D/9 [TCRC]; Uganda permit 004877 and USA permit 20US09881D/9 [NICS]). Research procedures at all three sanctuaries complied with Pan African Sanctuary Alliance standards and adhered to the American Society of Primatologists Principles for the Ethical Treatment of nonhuman Primates.

## Supporting information


**Figures S1‐S2.** Maximum‐likelihood phylogenetic trees of viruses identified in blood samples from sanctuary chimpanzees at TCS, TCRC, and NICS. Viruses identified in this study and Owens et al., 2021 are labeled by their abbreviation (see Table 1) and are marked with a colored star to indicate the sanctuary of origin (orange = TCS, purple = TCRC, blue = NICS). All other viruses are labeled by their host organism, country of origin, year of sample collection, and GenBank accession number. Statistical confidence in clades based on 1000 bootstrap replicates is represented by the numbers beside branches. The scale bar is equal to nucleotide substitutions per site.

Supplementary information.

## Data Availability

Viral nucleotide sequences are available in GenBank under accession numbers MT350347 to MT350357 and ON706335 to ON706349.
