# Supplementary material for: Viruses in sanctuary chimpanzees across Africa
Source: Am J Primatol. 2022 Nov 3;85(1):e23452. doi: 10.1002/ajp.23452 (PMC9812903; doi:10.1002/ajp.23452)
Supplement: Supplementary file 1 — Figures S1‐S2. Maximum‐likelihood phylogenetic trees of viruses identified in blood samples from sanctuary chimpanzees at TCS, TCRC, and NICS. Viruses identified in this study and Owens et al., 2021 are labeled by their abbreviation (see Table 1) and are marked with a colored star to indicate the sanctuary of origin (orange = TCS, purple = TCRC, blue = NICS). All other viruses are labeled by their host organism, country of origin, year of sample collection, and GenBank accession number. Statistical confidence in clades based on 1000 bootstrap replicates is represented by the numbers beside branches. The scale bar is equal to nucleotide substitutions per site. [file AJP-85-0-s001.pdf]

Supporting Information - Figures

Viruses in sanctuary chimpanzees across Africa

Emily Dunay, Leah A. Owens, Christopher D. Dunn, Joshua Rukundo, Rebeca Atencia, Megan F. Cole, Averill Cantwell, Melissa Emery Thompson, Alexandra G. Rosati, Tony L. Goldberg*

*Correspondence to Tony L. Goldberg (tony.goldberg@wisc.edu)

This document contains the following:

1. **Supporting Figures**
    a. **Figures S1 & S2:** Phylogenetic trees of viruses identified in sanctuary chimpanzees

Figure S1

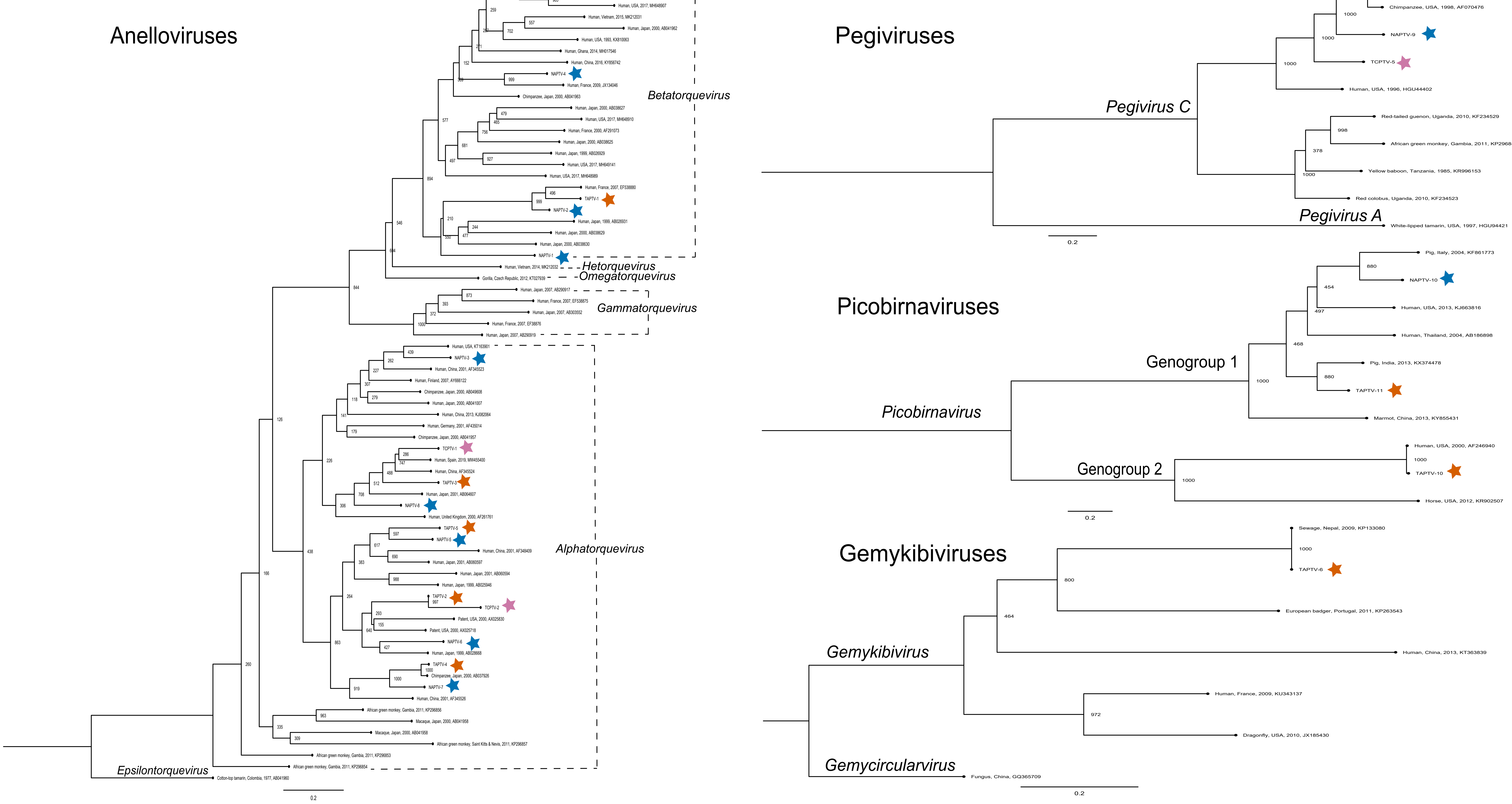
Anelloviruses
Betatorquevirus
Hetorquevirus
Omegatorquevirus
Gammatorquevirus
Alphatorquevirus
Epsilontorquevirus
0.2
Pegiviruses
Pegivirus C
Pegivirus A
TAPTV-8
Chimpanzee, USA, 1998, AF070476
NAPTV-9
TCPTV-5
Human, USA, 1996, HGU44402
Red-tailed guenon, Uganda, 2010, KF234529
Yellow baboon, Tanzania, 1985, KR996153
Red colobus, Uganda, 2010, KF234523
White-lipped tamarin, USA, 1997, HGU94421
0.2
Picobirnaviruses
Picobirnavirus
Genogroup 1
Genogroup 2
Pig, Italy, 2004, KF861773
NAPTV-10
Human, USA, 2013, KJ663816
Human, Thailand, 2004, AB186898
Pig, India, 2013, KX374478
TAPTV-11
Marmot, China, 2013, KY855431
Human, USA, 2000, AF246940
TAPTV-10
Horse, USA, 2012, KR902507
0.2
Gemykibiviruses
Gemykibivirus
Gemycircularvirus
Sewage, Nepal, 2009, KP133080
TAPTV-6
European badger, Portugal, 2011, KP263543
Human, China, 2013, KT363839
Human, France, 2009, KU343137
Dragonfly, USA, 2010, JX185430
Fungus, China, GQ365709
0.2

Figure S2

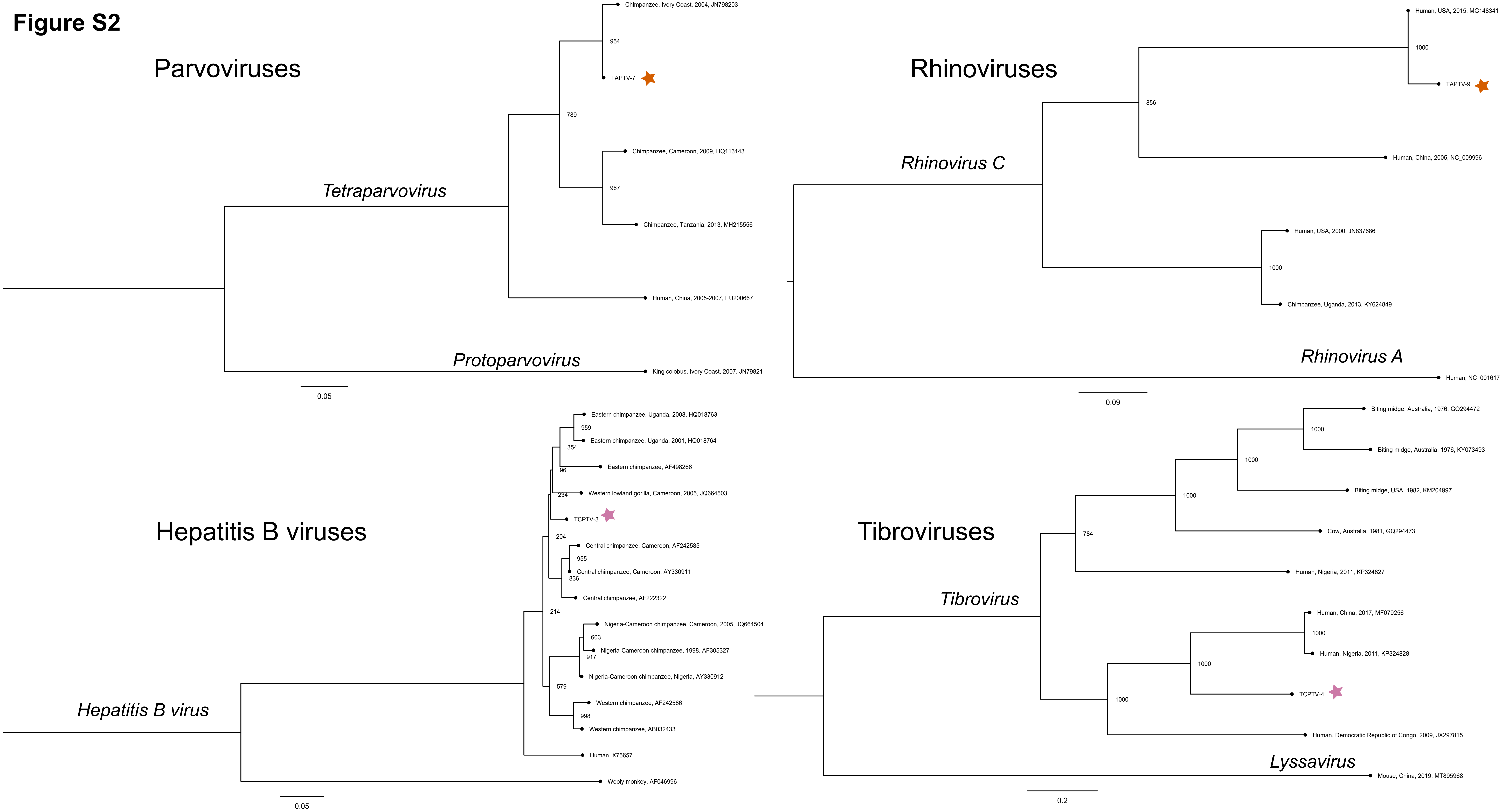
Parvoviruses
Chimpanzee, Ivory Coast, 2004, JN798203
954
TAPTV-7
789
Chimpanzee, Cameroon, 2009, HQ113143
967
Chimpanzee, Tanzania, 2013, MH215556
Tetraparvovirus
Human, China, 2005-2007, EU200667
Protoparvovirus
King colobus, Ivory Coast, 2007, JN79821
0.05
Rhinoviruses
Human, USA, 2015, MG148341
1000
TAPTV-9
856
Human, China, 2005, NC_009996
Rhinovirus C
Human, USA, 2000, JN837686
1000
Chimpanzee, Uganda, 2013, KY624849
Rhinovirus A
Human, NC_001617
0.09
Hepatitis B viruses
Eastern chimpanzee, Uganda, 2008, HQ018763
959
Eastern chimpanzee, Uganda, 2001, HQ018764
354
Eastern chimpanzee, AF498266
96
Western lowland gorilla, Cameroon, 2005, JQ664503
234
TCPTV-3
204
Central chimpanzee, Cameroon, AF242585
955
Central chimpanzee, Cameroon, AY330911
836
Central chimpanzee, AF222322
214
Nigeria-Cameroon chimpanzee, Cameroon, 2005, JQ664504
603
Nigeria-Cameroon chimpanzee, 1998, AF305327
917
Nigeria-Cameroon chimpanzee, Nigeria, AY330912
579
Western chimpanzee, AF242586
998
Western chimpanzee, AB032433
Human, X75657
Hepatitis B virus
Wooly monkey, AF046996
0.05
Tibroviruses
Biting midge, Australia, 1976, GQ294472
1000
Biting midge, Australia, 1976, KY073493
1000
Biting midge, USA, 1982, KM204997
1000
Cow, Australia, 1981, GQ294473
784
Human, Nigeria, 2011, KP324827
Tibrovirus
Human, China, 2017, MF079256
1000
Human, Nigeria, 2011, KP324828
1000
TCPTV-4
1000
Human, Democratic Republic of Congo, 2009, JX297815
Lyssavirus
Mouse, China, 2019, MT895968
0.2
