# Supplementary material for: Viruses in sanctuary chimpanzees across Africa
Source: Am J Primatol. 2022 Nov 3;85(1):e23452. doi: 10.1002/ajp.23452 (PMC9812903; doi:10.1002/ajp.23452)
Supplement: Supplementary file 2 — Supplementary information. [file AJP-85-0-s002.pdf]

Table S1. Detailed sample inventory for chimpanzees included in this study

Sanctuary	Chimpanzee	Sex	Age at time of sampling (years)	Sample type	Date collected
TCS	SLE02	F	9	Serum	02/03/16
	SLE05	F	12	Serum	02/06/16
	SLE07	M	12	Whole Blood	03/30/14
	SLE08	F	10	Serum	11/09/15
	SLE09	M	5	Serum	02/06/16
	SLE12	M	11	Serum	06/08/14
	SLE14	F	10	Serum	02/02/16
	SLE16	M	9.5	Serum	03/15/13
	SLE18	M	13	Plasma	05/30/16
	SLE23	F	12	Serum	03/06/15
	SLE24	F	14	Whole Blood in RNAlater	03/22/14
	SLE25	F	12	Spleen	07/11/16
	SLE26	F	13	Plasma	02/11/16
	SLE27	M	21	Serum	05/25/16
	SLE28	F	11	Serum	01/15/15
	SLE30	F	14	Serum	06/03/14
TCRC	ROC01	M	19	Plasma in DNA/RNA Shield	06/16/19
	ROC24	M	21	Plasma in DNA/RNA Shield	06/20/19
	ROC09	M	16.5	Plasma in DNA/RNA Shield	06/17/19
	ROC21	M	22	Plasma in DNA/RNA Shield	06/19/19
	ROC08	M	23	Plasma in DNA/RNA Shield	06/17/19
	ROC25	M	16.5	Plasma in DNA/RNA Shield	06/20/19
	ROC14	F	19	Plasma in DNA/RNA Shield	06/18/19
	ROC12	M	17	Plasma in DNA/RNA Shield	06/18/19
	ROC16	M	14	Plasma in DNA/RNA Shield	06/18/19
	ROC05	F	18	Plasma in DNA/RNA Shield	06/18/19
	ROC07	M	16.5	Plasma in DNA/RNA Shield	06/17/19
	ROC23	M	18	Plasma in DNA/RNA Shield	06/17/19
	ROC13	F	17	Plasma in DNA/RNA Shield	06/20/19
	ROC10	F	23	Plasma in DNA/RNA Shield	06/17/19
	ROC26	M	18	Plasma in DNA/RNA Shield	06/19/19
	ROC20	M	18	Plasma in DNA/RNA Shield	06/20/19
	ROC15	F	21	Plasma in DNA/RNA Shield	06/18/19
	ROC06	M	20	Plasma in DNA/RNA Shield	06/17/19
	ROC11	M	18	Plasma in DNA/RNA Shield	06/19/19
	ROC03[†]	M	20	Plasma in DNA/RNA Shield	06/16/19
	ROC02	F	19	Plasma in DNA/RNA Shield	06/16/19
	ROC17	M	18	Plasma in DNA/RNA Shield	06/18/19
	ROC18	M	22	Plasma in DNA/RNA Shield	06/19/19
	ROC22	F	19	Plasma in DNA/RNA Shield	06/19/19
	ROC19	F	19	Plasma in DNA/RNA Shield	06/19/19
NICS	UGA24	F	12	Plasma	07/25/16
	UGA42	M	19	Plasma	08/14/16
	UGA37	M	19	Plasma	08/20/16
	UGA08	F	27	Plasma	07/20/16
	UGA14	M	17	Plasma	07/22/16
	UGA25	M	20	Plasma	07/26/16
	UGA36	F	29	Plasma	08/10/16
	UGA02	F	32	Plasma	07/18/16
	UGA15	M	17	Plasma	07/22/16
	UGA03	M	32	Plasma	07/19/16
	UGA23	F	11	Plasma	07/25/16
	UGA01	F	32	Plasma	07/18/16
	UGA22	F	11	Plasma	07/25/16
	UGA45	F	15	Plasma	08/13/16
	UGA34	F	18	Plasma	08/14/16
	UGA38	F	15	Plasma	08/12/16
	UGA43	F	17	Plasma	08/14/16
	UGA28	F	20	Plasma	07/27/16
	UGA04	M	17	Plasma	07/19/16
	UGA30	F	17	Plasma	07/28/16
	UGA05	M	30	Plasma	07/19/16
	UGA13	M	12	Plasma	07/21/16
	UGA18	F	25	Plasma	07/23/16
	UGA33	F	7	Plasma	08/16/16
	UGA06	M	29	Plasma	07/20/16
	UGA26	M	19	Plasma	07/26/16
	UGA35	M	20	Plasma	08/15/16
	UGA31	F	18	Plasma	07/28/16

[†]Sanctuary-born

Table S2: Prevalence and univariate statistical associations between sex (male or female) and prevalence and load of viruses in chimpanzees at TCS, TCRC, and NICS.

			Prevalence (%)‡					Viral Load (log_{10}vRPM/kb)§			
	Virus ID†	Chimpanzees Infected (#)	Overall (n=16)	Male (n=6)	Female (n=10)	Odds Ratio	*p* value	Male (n=6)	Female (n=10)	U	*p* value
TCS	1	16	100 (77.3, 100)	100 (55.7, 100)	100 (67.9, 100)	0.62 (0.01, 35.17)	>0.9999	1.1771	1.1277	28	0.8749
	4	1	6.3 (<0.01, 30.3)	16.7 (1.1, 58.2)	0 (0, 3.2)	5.73 (0.20, 165.3)	0.2745	1.0116	NA	NA	NA
	6	2	12.5 (2.2, 37.3)	0 (0, 44.3)	20 (45.9, 52.1)	0.26 (0.01, 6.44)	>0.9999	NA	0.6418	NA	NA
	7	1	6.3 (<0.01, 30.3)	16.7 (1.1, 58.2)	0 (0, 3.2)	5.73 (0.20, 165.3)	0.2745	0.0859	NA	NA	NA
	9	4	25 (9.7, 50)	33.3 (9.3, 70.4)	20 (45.9, 52.1)	2 (0.20, 19.91)	0.978	0.8099	0.4797	2	1
	10	5	31.3 (13.9, 55.9)	33.3 (9.3, 70.4)	30 (10.3, 60.8)	1.17 (0.13, 10.22)	>0.9999	1.2735	0.8347	2	0.8
	11	5	31.3 (13.9, 55.9)	33.3 (9.3, 70.4)	30 (10.3, 60.8)	1.17 (0.13, 10.22)	>0.9999	1.0117	0.5693	1	0.4
	12	6	37.5 (18.4, 61.5)	16.7 (1.1, 58.2)	50 (23.7, 76.3)	0.2 (0.02, 2.39)	0.4301	2.3774	0.7477	0	0.333
	13	7	43.8 (23.1, 66.9)	50 (18.8, 81.2)	40 (16.7, 68.8)	1.5 (0.20, 11.53)	>0.9999	1.4375	1.1936	5	0.8571
	24	10	62.5 (38.5, 81.6)	50 (18.8, 81.2)	70 (39.2, 89.7)	0.43 (0.05, 3.48)	0.7832	1.056	1.9784	16	0.2667
	25	1	6.3 (<0.01, 30.3)	0 (0, 44.3)	10 (<0.01, 42.6)	0.49 (0.02, 13.92)	>0.9999	NA	0.773	NA	NA
	Virus ID†	Chimpanzees Infected (#)	Overall (n=25)	Male (n=17)	Female (n=8)	Odds Ratio	*p* value	Male (n=17)	Female (n=8)	U	*p* value
TCRC	2	16	64 (44.4, 79.8)	58.8 (36, 78.4)	75 (40.1, 93.7)	0.48 (0.07, 3.09)	0.7473	0.7894	0.1608	22	0.4378
	5	1	4 (<0.01, 21.1)	0 (0, 21.6)	12.5 (0.11, 49.2)	0.14 (0.01, 3.92)	>0.9999	NA	1.183	NA	NA
	14	3	12 (3.3, 30.8)	11.7 (2, 35.6)	12.5 (0.11, 49.2)	0.933 (0.07, 12.1)	>0.9999	0.7221	0.4738	1	1
	15	5	20 (8.4, 39.6)	17.7 (5.4, 41.8)	25 (6.3, 59.9)	0.64 (0.08, 4.89)	>0.9999	0.7178	0.355	1	0.4
	26	10	40 (23.4, 59.3)	41.2 (21.6, 64.1)	37.5 (13.5, 69.6)	1.17 (0.21, 6.56)	>0.9999	0.9527	0.2391	7	0.8889
	Virus ID†	Chimpanzees Infected (#)	Overall (n=28)	Male (n=12)	Female (n=16)	Odds Ratio	*p* value	Male (n=12)	Female (n=16)	U	*p* value
NICS	3	28	100 (85.7, 100)	100 (71.8, 100)	100 (77.3, 100)	0.76 (0.01, 40.87)	>0.9999	1.0681	1.2106	109	0.5676
	8	11	39.3 (23.5, 57.6)	33.3 (13.6, 61.2)	43.8 (23.1, 66.9)	0.64 (0.14, 3.04)	0.8708	0.6188	0.3511	13	0.9273
	16	6	21.4 (9.9, 39.9)	16.7 (3.5, 46)	25 (9.7, 50)	0.6 (0.09, 3.99)	0.9585	0.9681	0.3948	2	0.5333
	17	7	25 (12.4, 43.6)	33.3 (13.6, 61.2)	25 (9.7, 50)	1.5 (0.29, 7.81)	0.9449	0.5174	0.5869	10	0.6857
	18	12	42.9 (26.5, 61)	25 (8.3, 53.9)	56.3 (33.2, 76.9)	0.26 (0.05, 1.33)	0.2038	0.8343	0.5975	13	1
	19	13	46.4 (29.5, 64.2)	50 (25.4, 74.6)	43.8 (23.1, 66.9)	1.29 (0.29, 5.66)	>0.9999	0.5329	0.5115	23	0.8357
	20	27	96.4 (80.8, >99.9)	100 (71.8, 100)	93.8 (69.7, >99.9)	2.42 (0.09, 64.68)	0.6253	1.0832	1.1072	88	0.9427
	21	19	67.9 (49.2, 82.2)	58.3 (31.9, 80.7)	68.8 (44.2, 86.1)	0.64 (0.13, 3.03)	0.8607	0.7333	0.5902	39	0.7168
	22	16	57.1 (39.1, 73.5)	75 (46.2, 91.7)	43.8 (23.1, 66.9)	3.86 (4.07, 79.26)	0.2038	0.5921	0.7455	45	0.1738
	23	9	32.1 (17.8, 50.8)	41.7 (19.3, 68.1)	25 (9.7, 50)	2.14 (0.43, 10.74)	0.5971	0.4505	0.8316	13	0.5556

†Refers to Table 1.

‡Prevalence with 95% confidence intervals calculated using the modified Wald method, shown in parentheses. Odds ratios with 95% confidence intervals in parentheses. *P* values were calculated using Fisher's exact tests (two-tailed).

§Average viral load by sex with Mann-Whitney U statistics, and associated *p* values (infected chimpanzees only).

Table S3. Viral loads and richness of TCS chimpanzees

	Viral load (log_{10} vRPM/kb)											
	Virus†											
**Chimpanzee**	**1**	**4**	**6**	**7**	**9**	**10**	**11**	**12**	**13**	**24**	**25**	**All (Total)**	**Richness**
SLE02	3.551748	0	0	0	0.5515	1.207	0	1.6705	1.2524	2.0367	0	3.196657318	6
SLE05	0.293238	0	0	0	0	0	0	0.8218	0	1.7645	0	0.654002151	3
SLE07	0.226466	0	0	0	0	0	0	0	0	0	0	0.114008845	1
SLE08	0.027899	0	0	0	0	0.1409	0	0	0	3.4244	0	2.118429447	3
SLE09	0.024781	1.0116	0	0	0	0	1.1102	0	0	0.2513	0	0.315581207	4
SLE12	2.973542	0	0	0	0	0.477	0.9132	0	1.8769	0	0	2.620076188	4
SLE14	2.702785	0	0	0	0.4079	0	0	0	0	0.3567	0	2.346355833	3
SLE16	0.528688	0	0	0	0.1232	2.0701	0	2.3774	2.1693	2.6944	0	1.729944112	6
SLE18	3.212114	0	0	0.0859	1.4966	0	0	0	0.2664	0	0	2.856360614	4
SLE23	0.013383	0	0	0	0	0	0	0.1635	0	1.7149	0	0.548021189	3
SLE24	0.057062	0	0	0	0	0	0.5111	0	0	0	0	0.07565127	2
SLE25	0.131872	0	0.0429	0	0	0	0	0	0	0	0.7729	0.091393115	3
SLE26	0.159823	0	1.2406	0	0	0	0	0.1442	2.6205	1.6952	0	1.353497847	5
SLE27	0.097073	0	0	0	0	0	0	0	0	0.2223	0	0.057902187	2
SLE28	2.985087	0	0	0	0	1.156	1.067	0.9386	0.7221	2.8562	0	2.664427898	6
SLE30	1.354374	0	0	0	0	0	0.1298	0	0.1794	0	0	1.022055291	3

†Refers to Table 1

Table S4. Viral loads and richness of TCRC chimpanzees

	Viral load (log10 vRPM/kb)						
	Virus†						
**Chimpanzee**	**2**	**5**	**14**	**15**	**26**	**All (Total)**	**Richness**
ROC01	0.1077	0	0.4598	1.0436	0.1445	0.288844861	4
ROC24	0	0	0	0	3.1544	2.404452543	1
ROC09	0.0627	0	0	0	0	0.040012777	1
ROC21	0.5355	0	0	0	0	0.399945704	1
ROC08	0.3289	0	0	0	0	0.231484479	1
ROC25	0.0405	0	0	0	3.1075	2.357785641	2
ROC14	0	0	0	0	0	0	0
ROC12	0	0	0	0	0.0845	0.016237646	1
ROC16	0	0	0.9844	0.6062	0.4127	0.215585924	3
ROC05	0.3283	0	0	0	0	0.231026098	1
ROC07	0	0	0	0	0	0	0
ROC23	0.3178	0	0	0	0	0.222832354	1
ROC13	0.0442	0	0	0	0.3275	0.102452857	2
ROC10	0.2757	0	0.4738	0.5082	0.1506	0.248342937	4
ROC26	0	0	0	0	0	0	0
ROC20	0	0	0	0	0.2215	0.048416986	1
ROC15	0	0	0	0.2017	0	0.016646469	1
ROC06	0.2398	0	0	0	0.2541	0.203844221	2
ROC11	3.4425	0	0	0	0	3.23606087	1
ROC03	2.7605	0	0	0.5036	0	2.554581368	2
ROC02	0.077	1.1826	0	0	0	0.443661409	2
ROC17	0.0578	0	0	0	0.2427	0.086780833	2
ROC18	0	0	0	0	0	0	0
ROC22	0.1492	0	0	0	0	0.098581764	1
ROC19	0.0905	0	0	0	0	0.058458577	1

†Refers to Table 1

Table S5. Viral loads and richness of NICS chimpanzees

	Viral load (log10 vRPM/kb)											
	Virus†											
Chimpanzee	3	8	16	17	18	19	20	21	22	23	All (Total)	Richness
UGA24	2.0138	0.808	0	0	0	0	0.4913	0	0	0	1.630994	3
UGA42	3.4572	1.6748	0	0	0	0	0.7104	0	0.4741	0.32	3.064629	5
UGA37	1.224	0	0	0	0	0	1.186	0.3376	0.3931	0.4998	0.961578	5
UGA08	0.5127	0.4045	0	0	0	0	0.9363	0.3968	0.9088	0	0.508235	5
UGA14	0.498	0	0	0	0	0	0.8967	0	0	0	0.413285	2
UGA25	0.5817	0.2521	0	0.1609	0	0.1461	0.5845	0	0	0	0.40609	5
UGA36	1.1668	0.2904	0	0	0	0	1.0617	0.3241	0	0	0.888915	4
UGA02	1.0417	0.311	0	0	0	0	0.634	0	0.1787	0	0.737374	4
UGA15	1.4427	0.2807	0	0	0.2789	0	0.7724	0.6886	0	0.7172	1.11076	6
UGA03	1.4666	0	0	0	0	0	0.0847	0	0.1656	0	1.095692	3
UGA23	1.6698	0	0	0	0.155	0	0.6154	0	0	0	1.297119	3
UGA01	0.195	0	0	0	0	0	0	0	0	0	0.089473	1
UGA22	0.1624	0.1551	0.4577	0.173	0.7206	0.4387	0.4384	0	0.5448	1.7276	0.738496303	9
UGA45	0.2869	0	0	0	0.4118	0	1.1336	0.5414	0	0	0.466525	4
UGA34	1.2953	0	0	0	0.1291	0.276	0.8731	1.3614	0.7339	0.5433	1.044021	7
UGA38	0.7186	0	0	0	0.2324	0.1345	0.7283	0.7125	0	0	0.542362	5
UGA43	0.6213	0	0.2773	0.9289	1.3838	0.9302	2.2225	0.7377	0	1.3085	1.375318	8
UGA28	0.9414	0.2673	0.6723	1.0669	1.6832	1.3016	2.5498	0.7984	0	0	1.669235	8
UGA04	0.9344	0	1.6512	1.5815	2.0785	1.9562	2.9522	0.3728	0.4317	0	2.068838	8
UGA30	1.9391	0	0.172	0.1787	0.4426	0.3733	1.2286	0.378	0.7934	0.1202	1.579745	9
UGA05	0.6116	0	0	0	0	0.0791	0.6577	0.9934	0.6832	0.2651	0.534055	6
UGA13	1.5281	0	0	0.0896	0.1456	0	0.8768	0.5437	0.7243	0	1.184762	6
UGA18	1.6363	0	0	0	0	0	2.0129	0.3578	1.0258	0	1.467282	4
UGA33	1.1645	0	0	0	0	0	1.1154	0.7851	1.0329	0.4584	0.938028	5
UGA06	0.5856	0.2678	0.285	0.2378	0	0.5294	1.4247	1.5412	1.4059	0	0.927018	8
UGA26	0.3143	0	0	0	0	0.3185	1.7683	1.07	0.8882	0	0.932796	5
UGA35	0.1736	0	0	0	0	0.1683	1.084	0.319	0.1622	0	0.397874	5
UGA31	4.0036	0.221	0	0	0.2194	0.1262	0.5676	0.0995	0	0	3.609834	6

†Refers to Table 1

Table S6. Statistical comparisons of viral richness and total viral load Original

**Richness**			
**1) Kruskal-Wallis rank sum test**			
**Chi-squared**	**df**	***p* value**	
39.386	2	2.80E-09	
**2) Wilcoxon rank sum test**			
**Sanctuary 1**	**Sanctuary 2**	**Original *p* value**	**Adjusted *p* value (Benjamini-Hochberg)**
TCS	NICS	7.60E-03	7.60E-03
TCS	TCRC	2.30E-05	3.53-05
NICS	TCRC	1.10E-08	3.30E-08
**Total Viral Load (log_{10}vRPM/kb)**			
**1) Kruskal-Wallis rank sum test**			
**Chi-squared**	**df**	***p* value**	
17.091	2	0.00019	
**2) Wilcoxon rank sum test**			
**Sanctuary 1**	**Sanctuary 2**	**Original *p* value**	**Adjusted *p* value (Benjamini-Hochberg)**
TCS	NICS	0.7814	0.78
TCS	TCRC	0.0072	0.011
NICS	TCRC	4.30E-05	0.00013
